# Non-compaction is not a simple genetic disorder

**Published:** 2014-02

**Authors:** Josef Finsterer, Sinda Zarrouk-Mahjoub

**Affiliations:** Krankenanstalt Rudolfstiftung, Vienna, Austria; Laboratory of Biochemistry, UR Human Nutrition and Metabolic Disorders, Faculty of Medicine, Monastir, Tunisia

## Dear Sir

We read with interest the article by Osmonov *et al.* about an asymptomatic 16-year-old boy with left ventricular hypertrabeculation/non-compaction (LVHT) who was incidentally investigated cardiologically for repetitive monomorphic couplets/triplets of premature ventricular ectopic beats with left bundle branch block morphology and inferior QRS axis.^1^ We have the following comments and concerns.

We do not agree with the definition of LVHT as a genetic disorder. Although frequently associated with genetic disease, a clear-cut genotype/phenotype correlation has never been established for any of the mutated genes so far described in association with LVHT. An argument against a causal relationship is that in the majority of hereditary neuromuscular disorders (NMDs) associated with LVHT, LVHT is absent.^2^ Since the exact cause and pathomechanism of LVHT remains elusive, it is not justified to classify LVHT as a genetic disease.

The authors reported that systolic function improved after ablation. Did the patient also receive angiotensin converting enzyme inhibitors, angiotensin 2 blockers, beta-blockers or diuretics, or do the authors attribute improvement of systolic dysfunction within two months after the procedure exclusively to the ablation?

The authors mentioned that the boy was scheduled for plastic surgery. Which operation was the patient intended to undergo? Did the patient present with dysmorphism, any skin problems, or bone abnormalities, which are occasionally found in patients with LVHT?^3^ LVHT has not only been misdiagnosed as distal heterotrophic cardiomyopathy, dilated cardiomyopathy, or left ventricular apical thrombus, but has also been mixed up with aberrant bands, papillary muscles, apical type of hypertrophic cardiomyopathy, myocardial abscess and toxoplasmosis.^4^

LVHT frequently occurs familiarly.^5^ Were any other first-degree relatives investigated for LVHT? Did any of the first-degree relatives present with clinical cardiac disease? Was the family history positive for syncope, severe arrhythmias or sudden cardiac death?

LVHT is frequently associated with chromosomal aberrations or NMDs. Did the boy undergo cytogenetic investigations to confirm a chromosomal defect or was he ever investigated by a myologist to confirm or rule out NMD? When examining the patient cardiologically, did he present with myopathic face, weakness of the limb, axial or respiratory muscles, wasting, or with fasciculations? What was the level of serum creatine kinase and serum lactate?

We do not agree with the view that LVHT may develop into cardiomyopathy (discussion). LVHT is per se classified as an unclassified cardiomyopathy by the European and American Cardiological Society.

A follow up of two months is very short. It would be interesting to know about any long-term results. Did ventricular arrhythmias recur? Did left ventricular dysfunction or dilatation of the cardiac cavities re-emerge?

Overall, it would be helpful to receive more detailed information about the affected patient and his relatives to assess whether LVHT was associated with hereditary disease or not. Long-term data would help to assess whether the applied therapeutic measures truly had a long-term effect in this particular patient without developing arrhythmias other than ventricular ectopic beats or heart failure since then.
